# Supplementary figures and images for: Capn5 Expression in the Healthy and Regenerating Zebrafish Retina
Source: Invest Ophthalmol Vis Sci. 2018 Jul;59(8):3643–54. doi: 10.1167/iovs.18-24278 (PMC6054427; doi:10.1167/iovs.18-24278)

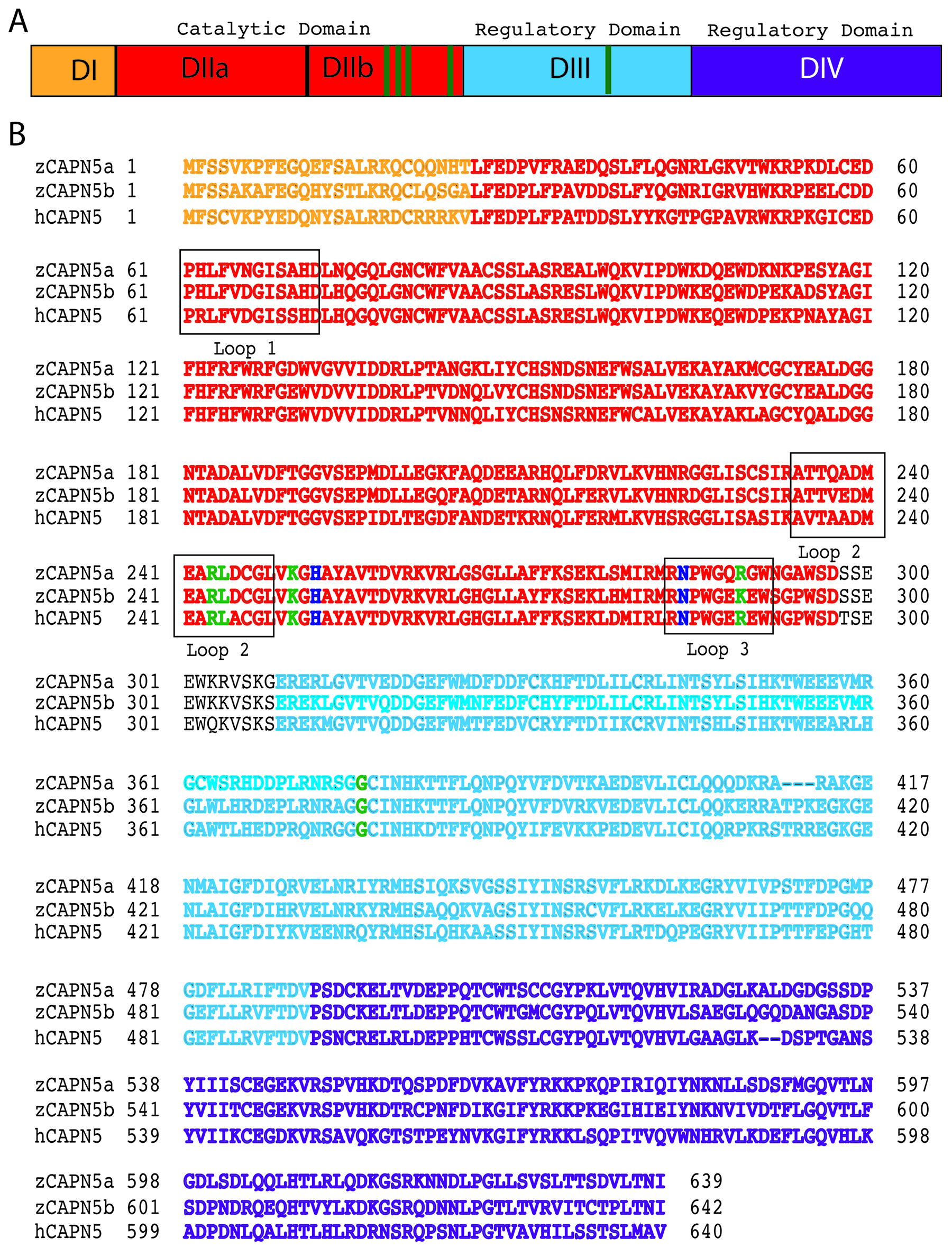

Supplement: Supplement 1 [file iovs-59-07-49_s01.tif]

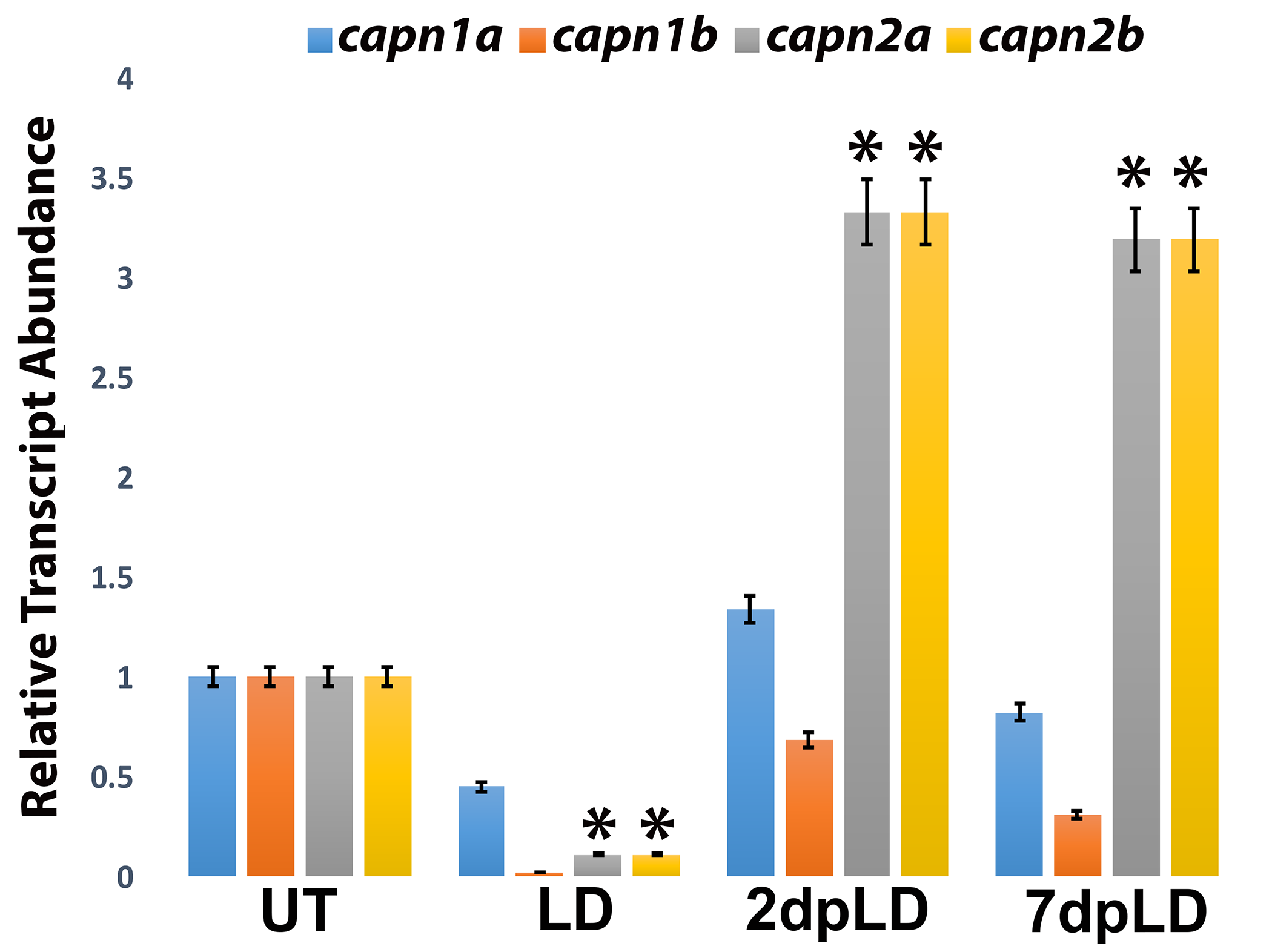

Supplement: Supplement 2 [file iovs-59-07-49_s02.tif]
