# Supplementary material for: Capn5 Expression in the Healthy and Regenerating Zebrafish Retina
Source: Invest Ophthalmol Vis Sci. 2018 Jul;59(8):3643–54. doi: 10.1167/iovs.18-24278 (PMC6054427; doi:10.1167/iovs.18-24278)
Supplement: Supplement 3 [file iovs-59-07-49_s03.pdf]

Table 1:

Gene	Forward Primer	Reverse Primer	Product Size	
capn1a	CTGGAGGATGAGGAAGAAA	TATCTAGAGTGAGGTTACAGCAGGA	637 bp	RT-PCR and Riboprobes
capn1b	CAGAAAGGCGAGTGGGTA	GAGCATCAGGAGTGAGGTTG	610 bp	RT-PCR and Riboprobes
capn2a	GCGAACGATTTTCAACA	CACATTTACACATTCCTGG	556 bp	RT-PCR and Riboprobes
capn2b	ACTTCTGCCTGCGTGTCTTC	ATTCTAGATTGAGTGCTGTGCTTTGA	650 bp	RT-PCR and Riboprobes
capn5a	TCCATCAGAGCCACAACTCAG	AGATGGAGCTGCCCACTGAT	652 bp	RT-PCR and Riboprobes
capn5b	CGCAGAGCTACTCCAAAGGAG	ACCCAGGAAGGTGTCAACAAT	558 bp	RT-PCR and Riboprobes
capn1a	GATAAAGATGGGAGCGACG	CTGATTCTACAGCGAGACGCA	148 bp	qPCR primers
capn1b	AGAAGGACAGAGCAACATTTC	ATAAAGGTCTCGGAGCGTGC	148 bp	qPCR primers
capn2a	GCTCTGTTGGGATGCTCCAT	GCACTCGGACCAGCTTTTCT	148 bp	qPCR primers
capn2b	AGCTCGGGAACCATAAGCAC	TCAGGCAGGACACGAAGTTG	148 bp	qPCR primers
capn5a	TGCCATCTCAGCAAAACTTG	CACAGGCTCAGGAACAGTCA	132 bp	qPCR primers
capn5b	TGTTTACAGCAGGCAGGACAA	ATGGAGCTGCCCACTGATTT	108 bp	qPCR primers
atp5h	TTTGAGGGGCAGCATTACTCC	CCCCTGGTAGAAGAGCGAATC	200 bp	RT-PCR and Riboprobes

Table 1. Primer sequences used for RT-PCR and qPCR. RT-PCR primers were also used to design WISH and FISH probes.
