# Supplementary material for: Capn5 Expression in the Healthy and Regenerating Zebrafish Retina
Source: Invest Ophthalmol Vis Sci. 2018 Jul;59(8):3643–54. doi: 10.1167/iovs.18-24278 (PMC6054427; doi:10.1167/iovs.18-24278)
Supplement: Supplement 4 [file iovs-59-07-49_s04.pdf]

Table 2.
Primary antibodies used in this study

| Antibody | Labels | Raised in | Dilution | Acquired from |
|---|---|---|---|---|
| Zpr-1 | red/green cones | mouse | 1:20 | ZIRC |
| 4C12 | rods | mouse | 1:100 | James Fadool FSU, Tallahassee, FL |
| Zrf-1 | Müller glia | mouse | 1:5000 | ZIRC |
| N1C1 | Capn5 | rabbit | 1:100 | GeneTex |
| GFP | GFP | rabbit | 1:1000 | abcam |

**Table 2.** Antibodies used for immunohistochemistry (IHC).
