# Supplementary material for: Capn5 Expression in the Healthy and Regenerating Zebrafish Retina
Source: Invest Ophthalmol Vis Sci. 2018 Jul;59(8):3643–54. doi: 10.1167/iovs.18-24278 (PMC6054427; doi:10.1167/iovs.18-24278)
Supplement: Supplement 5 [file iovs-59-07-49_s05.pdf]

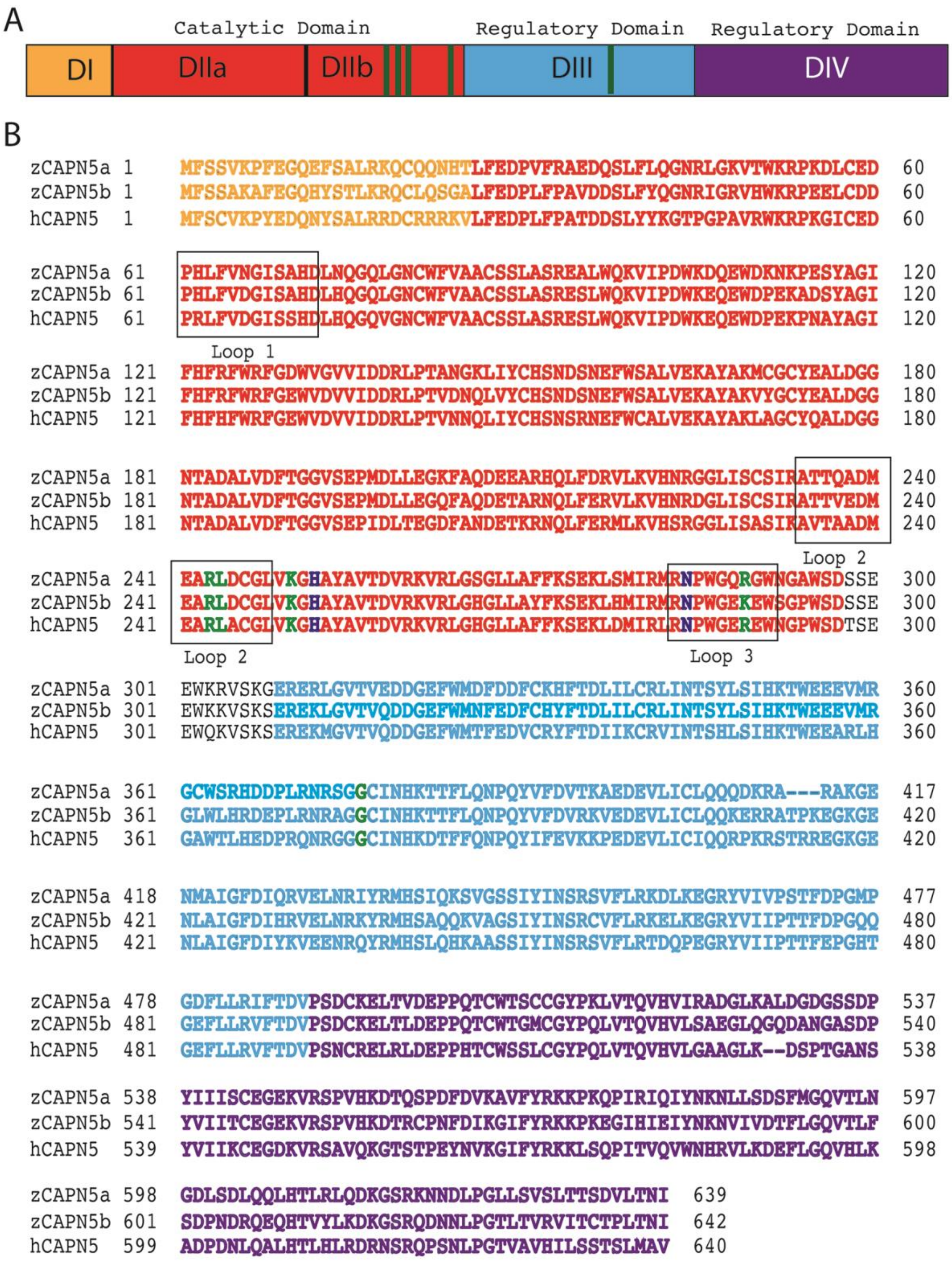


**Supplemental figure 1. High level of conservation among zebrafish orthologs of CAPN5.** (A) CAPN5 is composed of four domains; DI (yellow), DII (red), DIII (light blue) and DIV (purple). Four of the identified mutations are location in DII (green) and one in

DIII. (B) Primary Protein alignment of the zebrafish CAPN5 orthologs and human CAPN5 shows high conservation in the catalytic residues (blue) as well as the identified mutations (green). High conservation can also be seen amongst the gated loops of DII (black boxes).

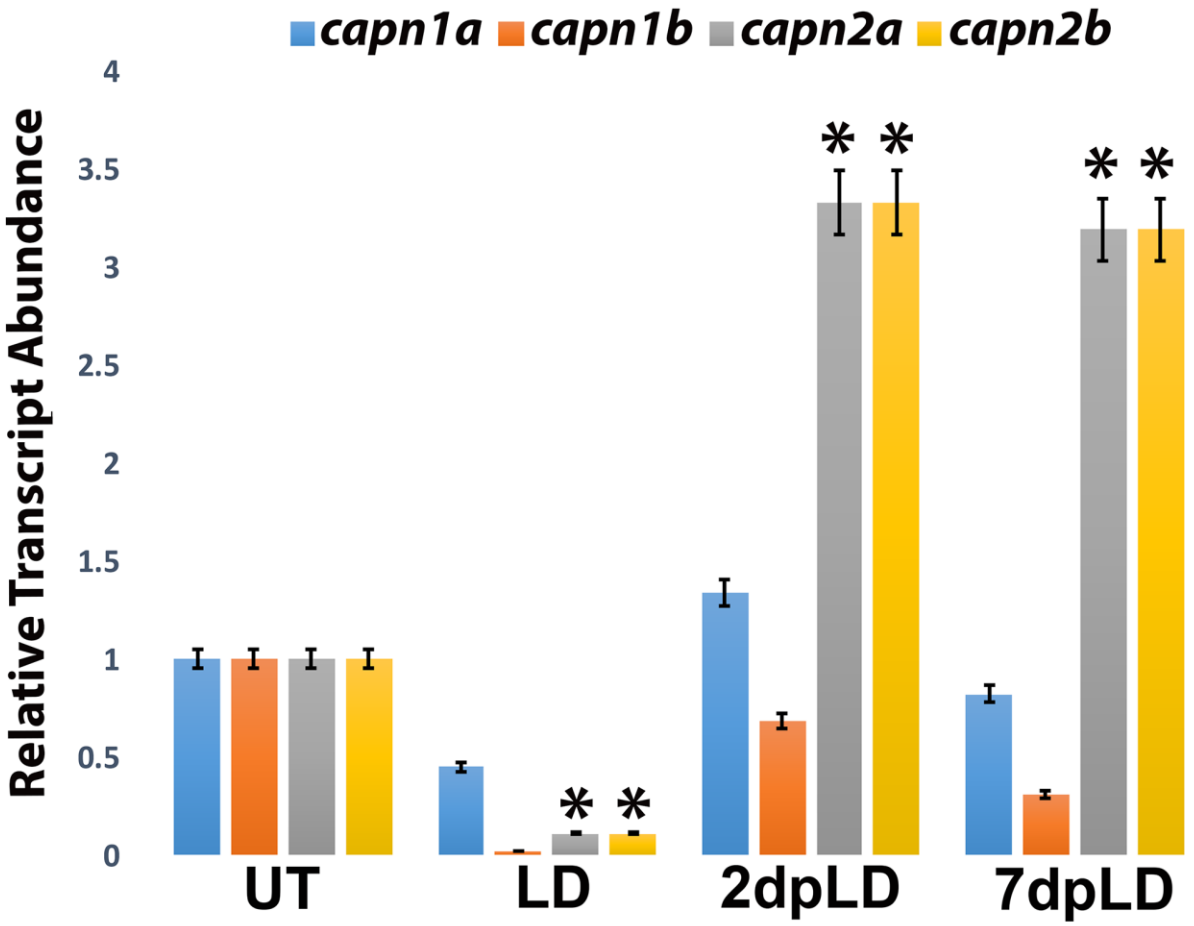


**Supplementary Figure 2. Expression of *capn1* and *capn2* in response to acute light damage.** qPCR for *capn1a/b* and *capn2a/b* during and following light damage. A significant decrease in expression of *capn2a/b* was observed during light damage and a significant increase was observed after light damage. *Capn1a/b* expression did not change during LD. RT, reverse transcriptase; NT, no template control; p>0.05 (*).

**Supplemental Table 1.** Primer sequences used for RT-PCR and qPCR. RT-PCR primers were also used to design WISH and FISH probes.

**Supplemental Table 2.** Antibodies used for immunohistochemistry (IHC).
